# Supplementary material for: APOBEC3B drives PKR-mediated translation shutdown and protects stress granules in response to viral infection
Source: Nat Commun. 2023 Feb 14;14:820. doi: 10.1038/s41467-023-36445-9 (PMC9925369; doi:10.1038/s41467-023-36445-9)
Supplement: Supplementary file 1 — Supplementary Information [file 41467_2023_36445_MOESM1_ESM.pdf]

Supplementary information for

APOBEC3B drives PKR-mediated translation shutdown and protects stress granules in response to viral infection

Lavanya Manjunath, Sunwoo Oh, Pedro Ortega, Alexis Bouin, Elodie Bournique, Ambrocio Sanchez, Pia Møller Martensen, Ashley A. Auerbach, Jordan T. Becker, Marcus Seldin, Reuben S. Harris, Bert L. Semler, & Rémi Buisson

Supplementary Figure 1

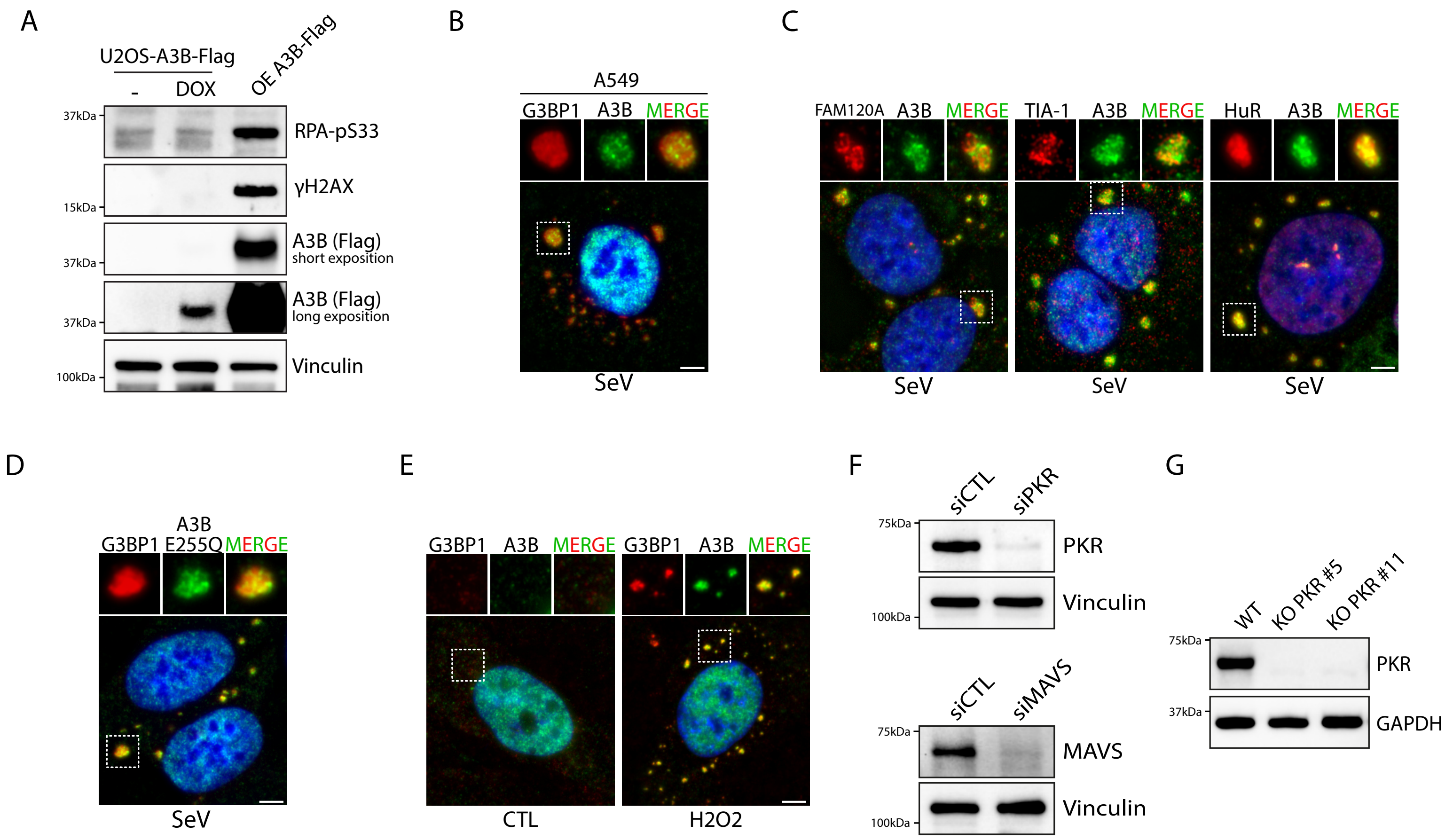

Supplementary Figure 1: A. The levels of DNA damage were analyzed by western blot using indicated antibodies in U2OS-A3B-Flag ±DOX or in U2OS cells that were transiently transfected with a vector expressing A3B-Flag. OE: Over Expressed **B.** A549-A3B-Flag treated with DOX were infected with SeV (MOI=1, 24hpi) and A3B and G3BP1 localization were monitored by immunofluorescence with a flag and a G3BP1 antibody respectively. Scale bar: 5 µm. **C.** U2OS-A3B-Flag treated with DOX were infected with SeV (MOI=1, 24hpi) and A3B co-localization with the indicated SG markers was monitored by immunofluorescence. Scale bar: 5 µm. **D.** The localization of A3B-E255Q-Flag to SGs after SeV infection at MOI=1 was monitored by immunofluorescence at 24hpi. Scale bar: 5 µm. **E.** Immunofluorescence of A3B (Flag) and G3BP1 in U2OS-A3B-flag treated with DOX and H_2O_2 (2µM, 2h). Scale bar: 5 µm. **F.** U2OS cells were knockdown for PKR or MAVS for 40h and proteins extracts were analyzed by western blot using the indicated. **G.** PKR KO cells were analyzed by western blot using PKR antibody. Source data are provided as a Source Data file.

Supplementary Figure 2

A

~100-130 kDa sample				
Total peptides		Unique peptides		Gene
Replicate 1	Replicate 2	Replicate 1	Replicate 2	
127	99	27	30	HNRNPU (SAF-A)
125	126	33	33	MATR3
76	47	39	30	XRN2
56	28	27	19	CEP97
33	20	29	19	MOV10
33	22	25	21	DHX36

~60-75k kDa sample				
Total peptides		Unique peptides		Gene
Replicate 1	Replicate 2	Replicate 1	Replicate 2	
150	455	34	54	DDX3 (X/Y)
467	105	44	26	PABPC1
14	132	10	34	ZNF326
125	119	33	31	HSPA8
46	78	22	29	DDX17
20	54	17	31	HNRNPM
36	48	20	31	HSPA5
32	37	25	26	HSPA9
18	16	18	15	HNRNPQ

B

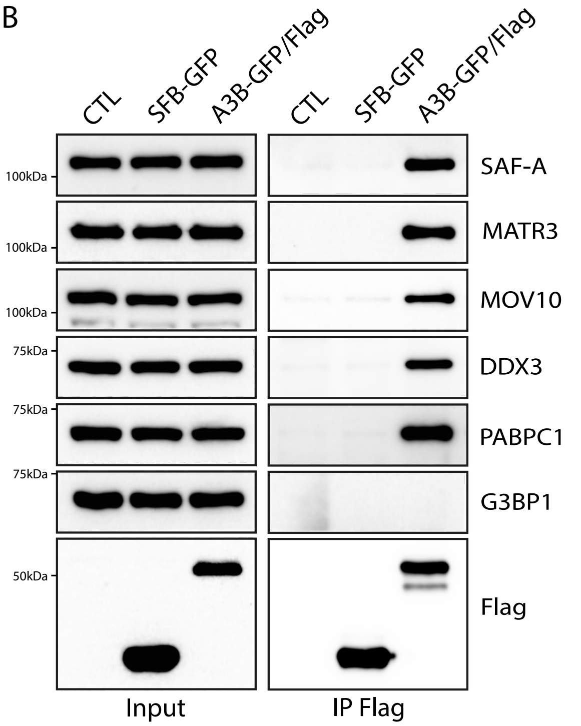

C

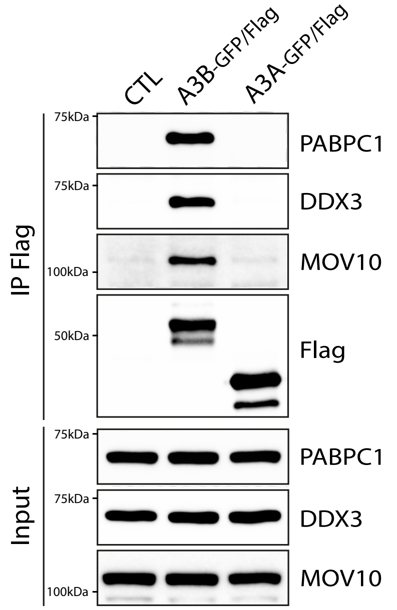

D

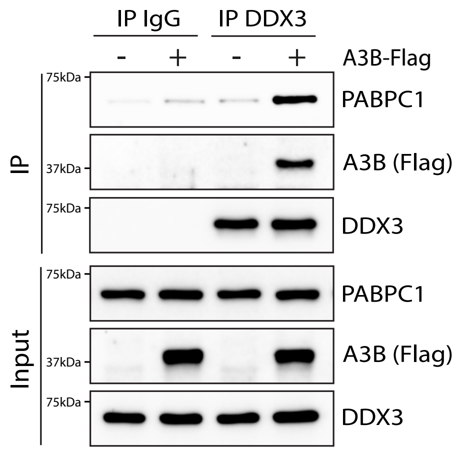

E

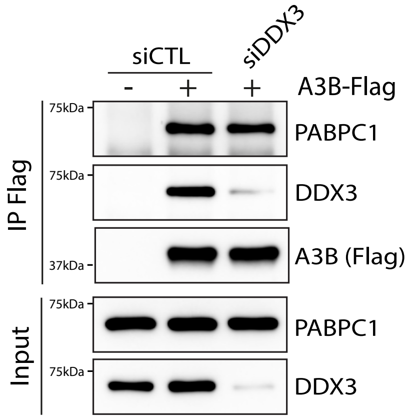

F

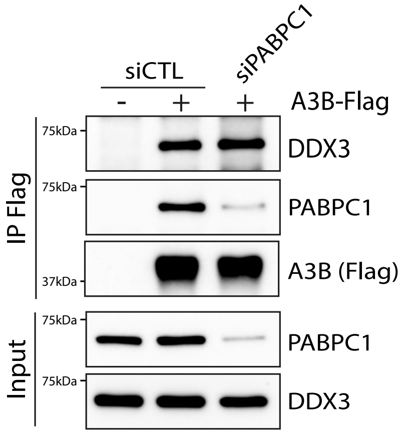

Supplementary Figure 2: A. Top interacting proteins from Mass Spectrometry analysis of A3B pull down samples from HEK-293T cells at the indicated molecular weight. **B.** Immunoprecipitation of A3B-GFP/Flag or of SFB-GFP in U2OS were analyzed by western blot with the indicated antibodies. **C.** A3B-GFP/Flag and A3A-GFP/Flag were transiently transfected in U2OS cells and immunoprecipitated using Flag antibody. The interactions between the indicated proteins and A3B or A3A were analyzed by western blot. **D.** Immunoprecipitation of DDX3 from U2OS-A3B-flag cells ± DOX and analyzed by western blot with the indicated antibodies. **E-F.** Immunoprecipitation of A3B-Flag from U2OS-A3B-flag cells ± DOX and knocked down for DDX3 (**E**) or PABPC1 (**F**) for 40h. The interaction levels between A3B, PABPC1 add DDX3 were analyzed by western blot. Source data are provided as a Source Data file.

Supplementary Figure 3

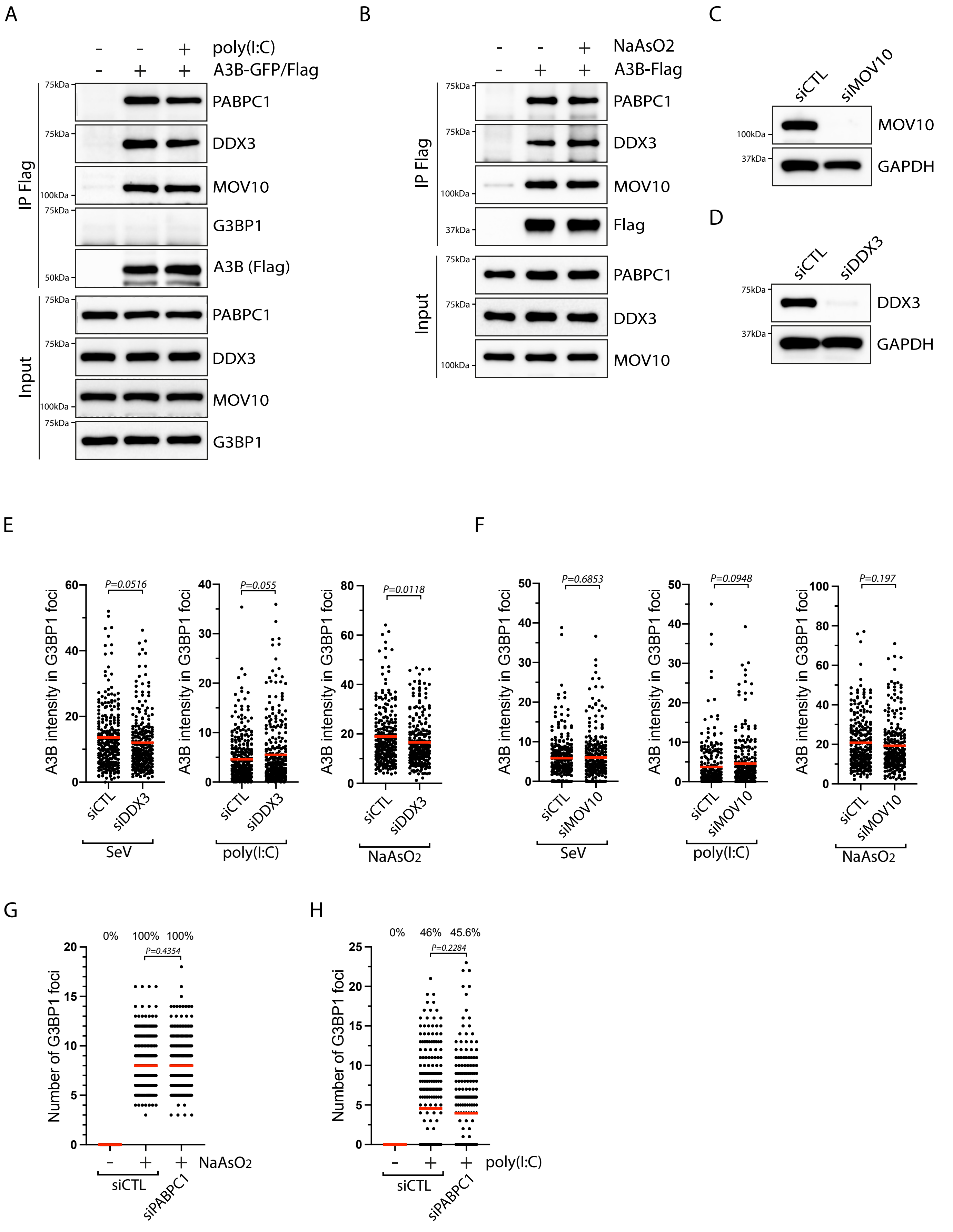

Supplementary Figure 3: A-B. Immunoprecipitation of A3B-Flag from U2OS-A3B-flag cells ± DOX transfected with poly(**I**:C) (200 ng/mL, 16h) (**A**) or treated with $NaAsO_2$ (500 µM, 1h) (**B**) followed by western blot using indicated antibody. **C-D.** U2OS cells transfected with siRNA control or against MOV10 (**C**) or DDX3 (**D**) for 40h or followed by analysis of protein extracts by western blot using the indicated antibodies. **E-F.** Quantification of A3B intensity (arbitrary units) in G3BP1 foci in U2OS-A3B-flag cells treated with DOX and transfected with siRNA control (siCTL) or against DDX3 (**E**) or MOV10 (**F**) for 40h and then infected with SeV (MOI=1, 24hpi, Number of G3BP1 foci, n=250), transfected with poly(**I**:C) (200 ng/mL, 16h, Number of G3BP1 foci, n=300) or treated with NaAsO2 (250 µM, 1h, Number of G3BP1 foci, n=250). Red lines indicate the mean. P-values were calculated with a two-tailed Welch t-test. **G-H.** Quantification of the number of G3BP1 foci in individual U2OS cells transfected with the indicated siRNAs for 40h and treated with NaAsO2 (500 µM, 1h) (**G**) or transfected with poly(**I**:C) (200 ng/mL, 16h) (**H**). Top; percentage of cells with G3BP1 foci. Red lines indicate the mean (Number of cells, n=250). P-values were calculated with a two-tailed Welch t-test. Source data are provided as a Source Data file.

Supplementary Figure 4

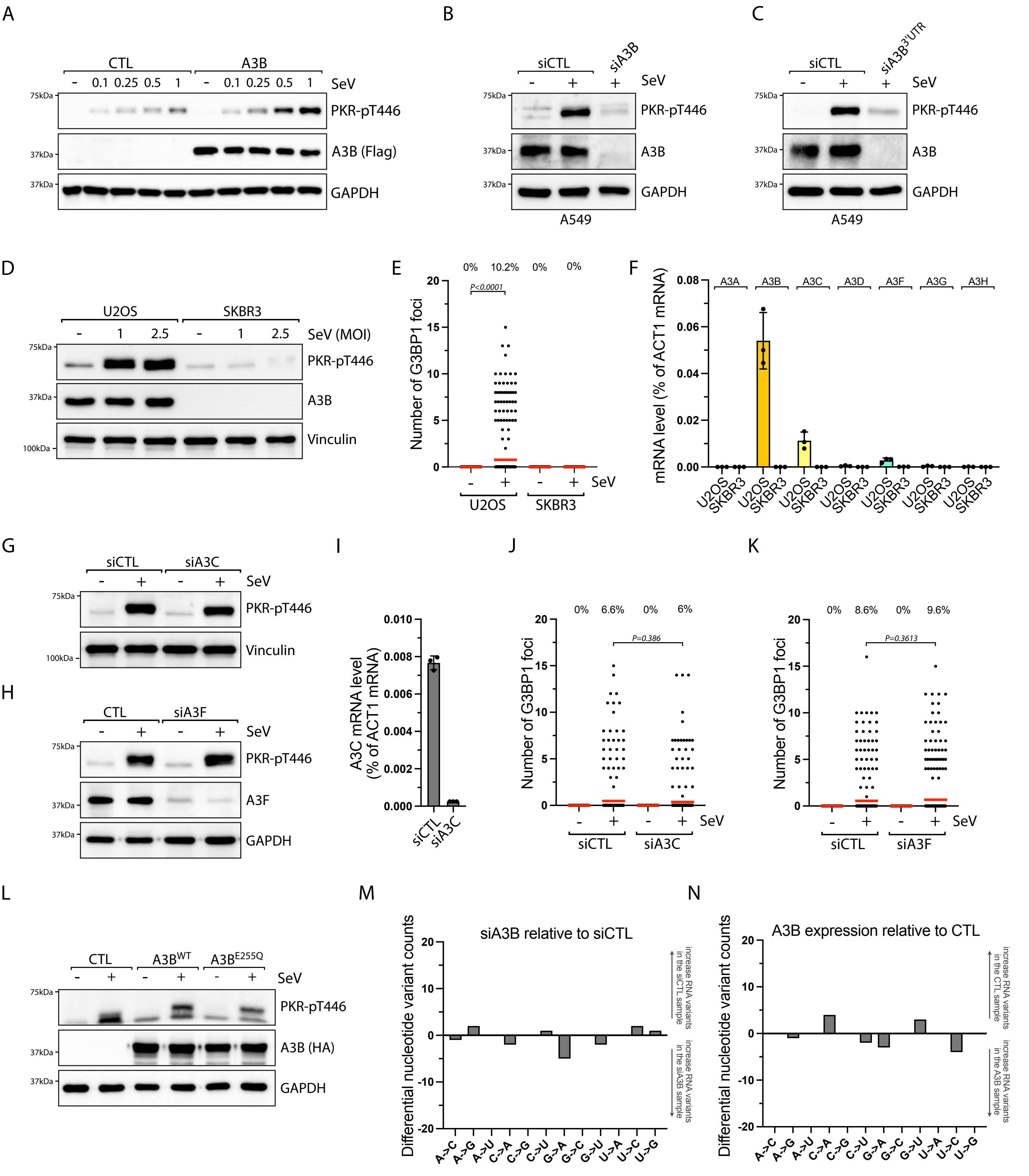

Supplementary Figure 4: A. U2OS-A3B-Flag ± DOX were infected with SeV of indicated MOI. At 24hpi the levels of PKR-pT446, A3B, and PKR were analyzed by western blot. **B-C.** A549 cells were transfected with siRNA control or siRNAs against A3B (**B**) or A3B-3'UTR (**C**) and infected with SeV (MOI=1, 24hpi). The levels of PKR-pT446, A3B, and GAPDH were analyzed by western blot. **D.** U2OS and SKBR3 cells were infected with SeV at the indicated MOI. The levels of PKR-pT446 and Vinculin were detected by western blot 24hpi. **E.** Quantification of the number of G3BP1 foci in individual U2OS and SKBR3 cells infected with SeV (MOI=1, 24hpi). Top; percentage of cells with G3BP1 foci. Red lines indicate the mean (Number of cells, n=500). P-values were calculated with a two-tailed Welch t-test. **F.** Expression level of the indicated genes were determined by RT-qPCR in U2OS and SKBR3 cells. Mean values ± SD (Number of technical replicates, n=3). **G-H.** The level of PKR-pT446 in U2OS cells transfected with siCTL, siA3C (**G**), or siA3F (**H**) for 40h followed by SeV (MOI=1, 24hpi) was analyzed by western blot**. I.** The A3C mRNA level was monitored in U2OS cells transfected with siCTL or siA3C for 40h. Mean values ± SD (Number of biological replicates, n=3). **J-K.** The number of G3BP1 foci by cell were quantified in U2OS cells transfected with siCTL, siA3C (**J**), or siA3F (**K**) for 40h followed by SeV infection (MOI=1, 24hpi). Top; percentage of cells with G3BP1 foci. Red lines indicate the mean (Number of cells, n=500). P-values were calculated with a two-tailed Welch t-test. **L.** U2OS cells were transfected with A3B-WT or A3B-E255Q and infected with SeV (MOI=1, 24hpi). The level of PKR-pT446 was analyzed by western blot. **M.** RNA isolated from U2OS cells transfected with siCTL or siA3B followed by SeV infection (MOI=1, 24hpi) was sequenced and aligned to SeV genome sequence to monitor RNA editing events. The number of mutations for each nucleotide variant across SeV genome was called by GATK's Mutect2 variant caller directly from aligned BAM files from RNA-Seq, where a minimal threshold was used to retain all possible sequence variants. Specific nucleotide mutations are shown on the x-axis and the ratio of A3B siRNA are plotted relative to control sample (y-axis). Only point mutations present in three or more sequencing reads at a particular position and not present in all sequencing reads were selected as RNA editing events. Positive bar graph indicates more mutations for the specific nucleotide variant in siCTL sample while negative bar graph indicates more mutations in siA3B sample. **N.** The number of mutations in SeV genome was determined in U2OS cells ± DOX infected with SeV (MOI=1, 24hpi). Positive bar graph indicates more mutations for the specific nucleotide variant in control sample while negative bar graph indicates more mutations in A3B-overexpressed sample. Source data are provided as a Source Data file.

Supplementary Figure 5

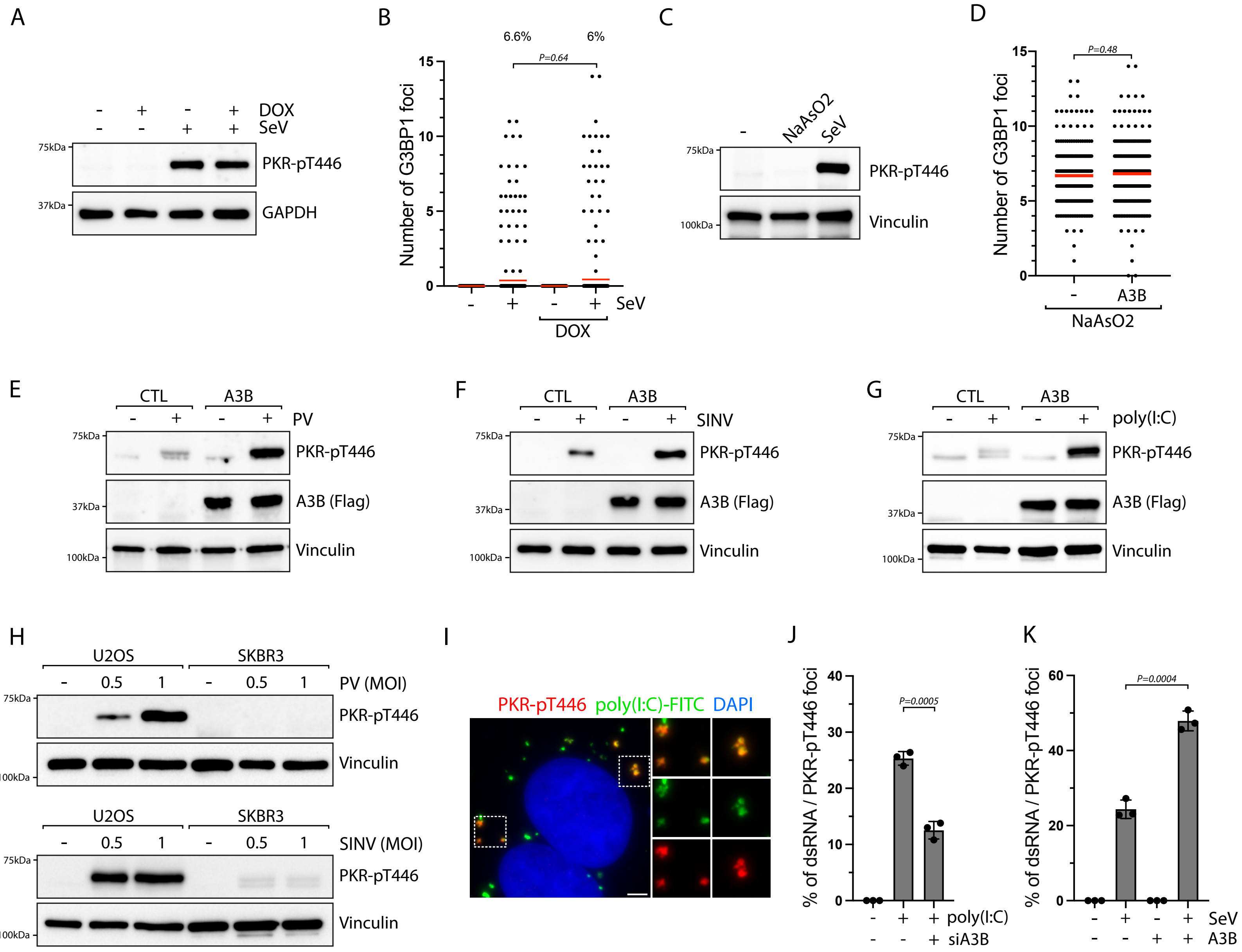

Supplementary Figure 5: A. U2OS WT cells ± DOX were infected with SeV (MOI=1, 24hpi) and levels of PKR-pT446, and Vinculin were analyzed by western blot. **B.** Quantification of number of G3BP1 foci in individual U2OS WT cells ± DOX and infected with SeV (MOI=1, 24hpi). Red lines indicate the mean (Number of cells, n=500). P-values were calculated with a two-tailed Welch t-test. **C.** U2OS cells were treated with $NaAsO_2$ (500 µM, 1h) or infected with SeV (MOI=1, 24hpi) and analyzed by western blot using the indicated antibodies. **D.** Quantification of number of G3BP1 foci by cell in U2OS-A3B-Flag ± DOX and treated with $NaAsO_2$ (250 µM, 1h). Red lines indicate the mean (Number of cells, n=100). P-values were calculated with a two-tailed Welch t-test. **E-G.** U2OS-A3B-Flag cells ± DOX were infected with PV (MOI=0.5, 24hpi) (**E**), SINV (MOI=1, 24hpi) (**F**), or transfected with poly(I:C) (200 ng/mL, 16h) (**G**) and analyzed by western blot using antibodies against the indicated proteins. **H.** U2OS and SKBR3 cells were infected with SINV or PV (MOI=0.5 and 1, 24hpi). The levels of PKR-pT446 and Vinculin were detected by western blot **I.** Immunofluorescence of PKR-pT446 and dsRNA (J2) in U2OS cells transfected with poly(I:C)-FITC (200 ng/ml, 4h). Scale bar: 5 µm. **J.** Quantification of percentage of cells with PKR-pT446 aggregate colocalizing with poly(I:C) in U2OS cells transfected with the indicated siRNAs for 40h and poly(I:C)-FITC (200 ng/ml, 4h). Mean values ± SD (Number of biological replicates, n=3). P-values were calculated with a two-tailed Welch t-test. **K.** Quantification of percentage of cells with PKR-pT446 aggregate colocalizing with poly(I:C) in U2OS-A3B-Flag cells ± DOX transfected with poly(I:C)-FITC (200 ng/ml, 4h). Mean values ± SD (Number of biological replicates, n=3). P-values were calculated with a two-tailed Welch t-test. Source data are provided as a Source Data file.

Supplementary Figure 6

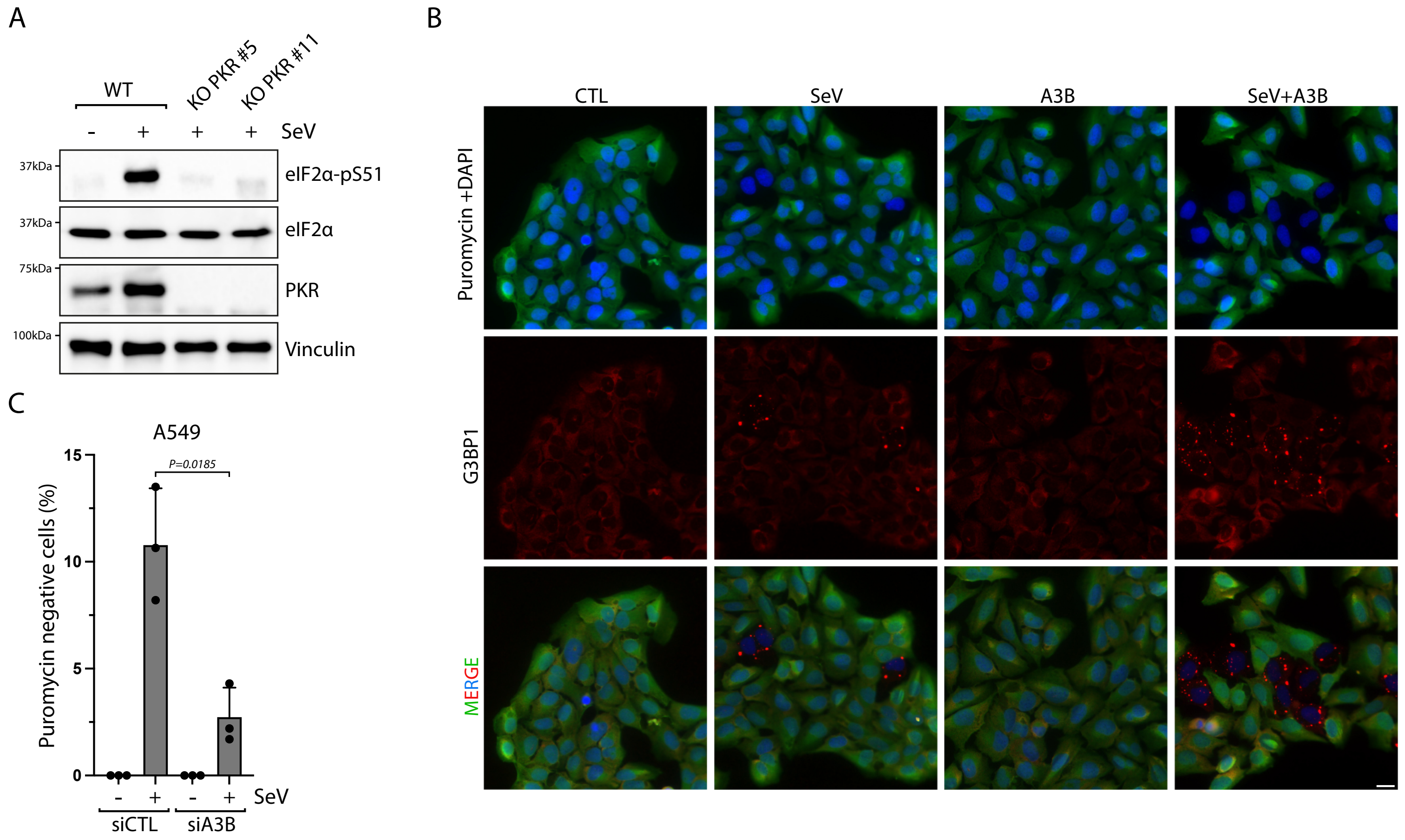

Supplementary Figure 6: A. U2OS-A3B-Flag and U2OS-A3B-Flag KO PKR cells without DOX treatment were infected with SeV (MOI=1, 24hpi) and analyzed by western blot with the indicated antibodies. **B.** Representative immunofluorescence of G3BP1 and Puromycin in U2OS-A3B-Flag cells ± DOX and/or infected with SeV (MOI=1, 24hpi). Scale bar: 20 µm **C.** Quantification of puromycin negative cells (%) in A549 cells transfected with siRNA control (siCTL) or siA3B for 40h and subsequently infected with SeV (MOI=1, 24hpi). Mean values ± SD (Number of biological replicates, n=3). P-values were calculated with a two-tailed Welch t-test. Source data are provided as a Source Data file.

Supplementary Figure 7

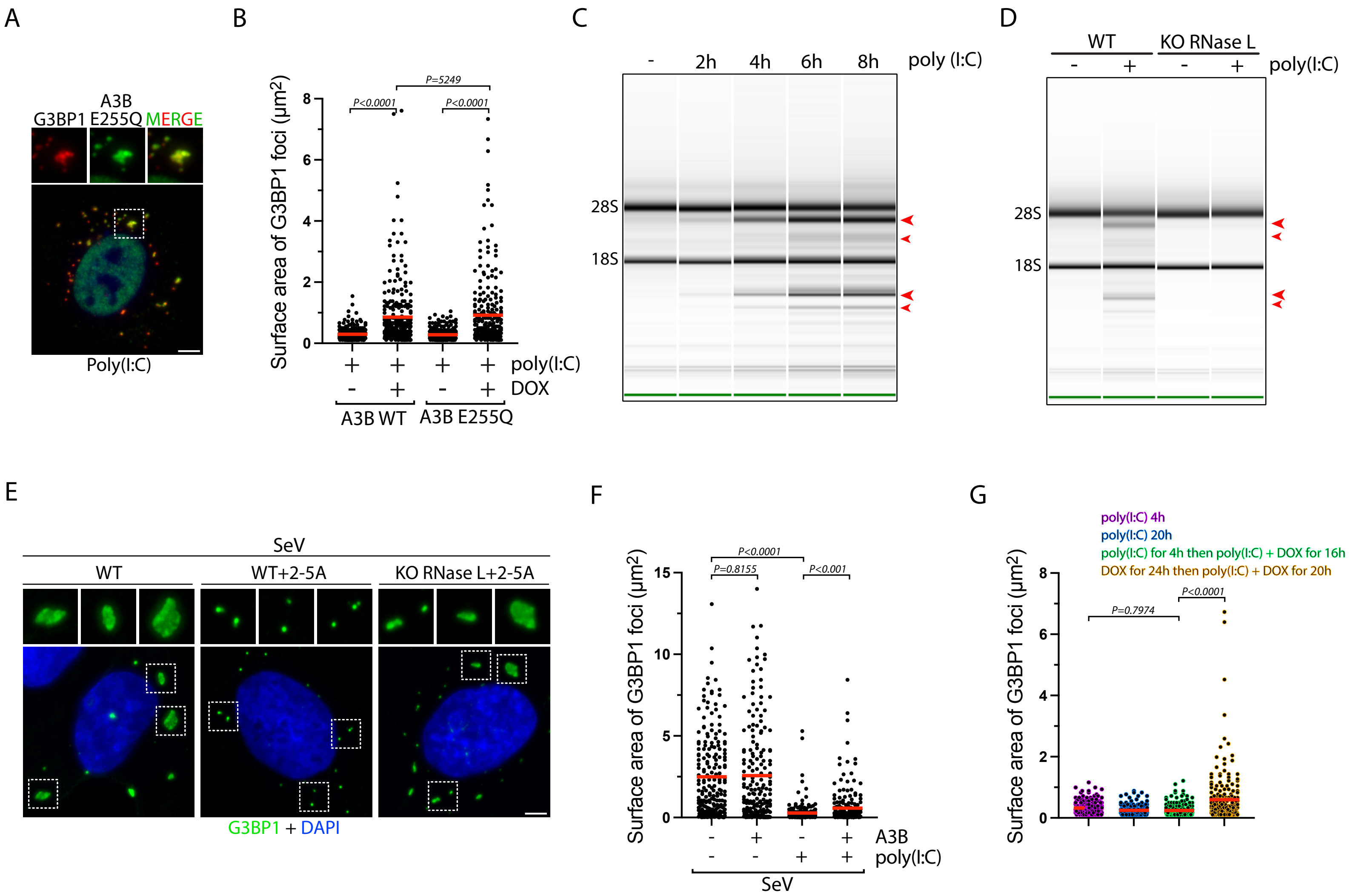

Supplementary Figure 7: A. The localization of A3B-E255Q-Flag to SGs was monitored by immunofluorescence 16h after transfection with poly(I:C) (200 ng/mL). Scale bar: 5 µm. **B.** Quantification of 250 G3BP1 foci surfacec form U2OS cells expressing the indicated constructs and transfected with poly(I:C) (200 ng/ml, 16h). Red lines indicate the mean. P-values were calculated with a two-tailed Welch t-test. **C.** U2OS cells transfected with poly(I:C) (400 ng/ml) were collected at different time point and total RNA was analyzed by bioanalyzer. The red arrows indicated ribosomal RNA cleavage products. **D.** Total RNA from U2OS-A3B-Flag WT and RNase L KO cells transfected with poly(I:C) (400 ng/mL, 8h) without DOX treatment were collected and analyzed for RNA integrity**.** **E.** Formation of G3BP1 foci after 2-5A transfection (20 µM, 16h) was monitored by immunofluorescence in U2OS-A3B-Flag WT and RNase L KO cells without DOX treatment. Scale bar: 5 µm**.** **F.** Quantification of 200 G3BP1 foci surface area from U2OS-A3B-Flag cells infected with SeV for 24h then transfected with poly(I:C) (200 ng/mL, 16h). Red lines indicate the mean. P-values were calculated with a two-tailed Welch t-test. **G.** Quantification of 200 G3BP1 foci surface area from U2OS-A3B-Flag cells with the indicated treatment. Red lines indicate the mean. P-values were calculated with a two-tailed Welch t-test.

Supplementary Figure 8

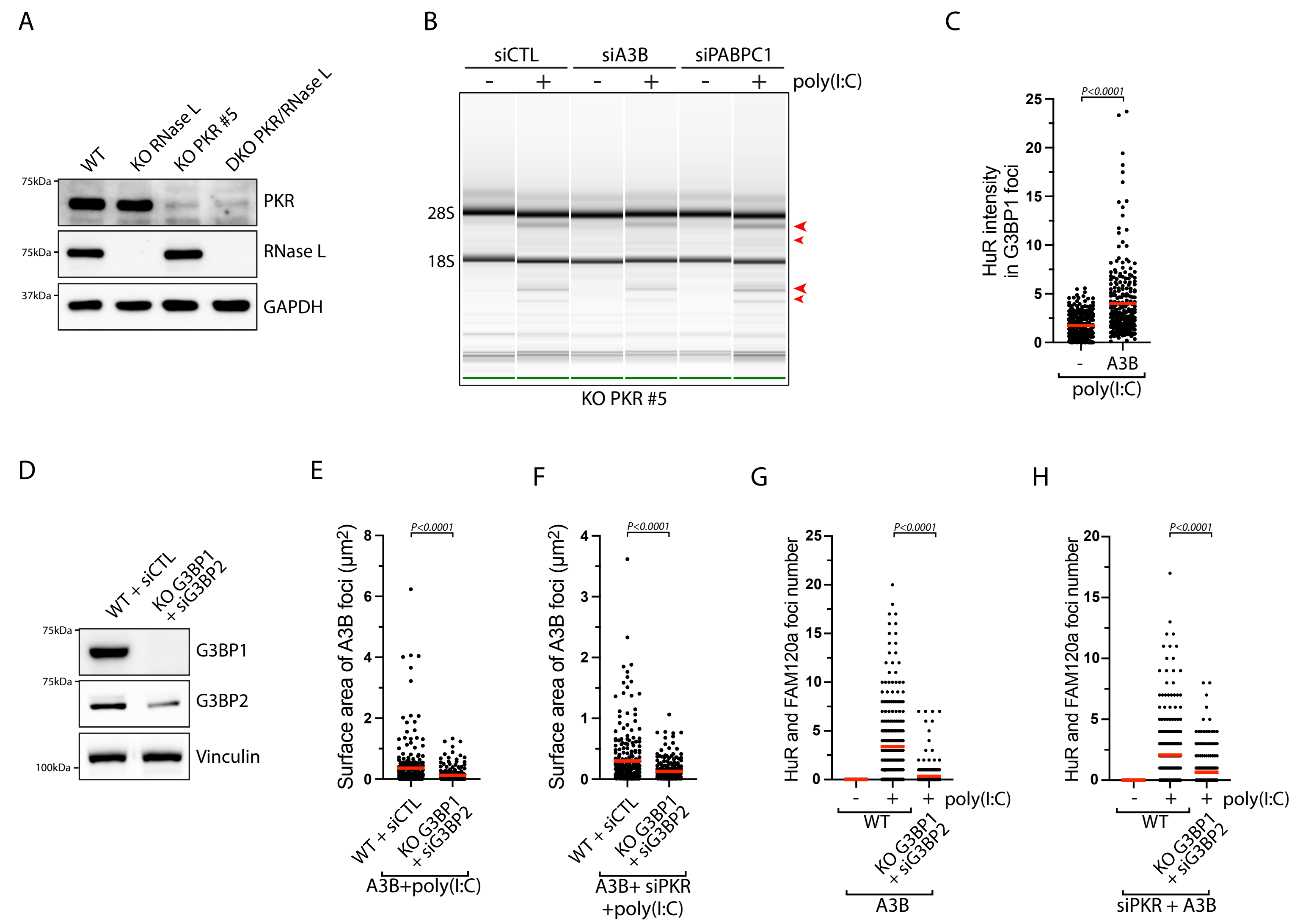

Supplementary Figure 8: A. The level of PKR, RNase L, and GAPDH were analyzed in the indicated cell lysates by western blotting. **B.** U2OS PKR KO cells transfected with siRNAs for 40h and/or poly(I:C) (200 ng/ml) were collected at 4h and total RNA was analyzed by bioanalyzer. The red arrows indicated ribosomal RNA cleavage products. **C.** Quantification of HuR intensity (arbitrary units) in G3BP1 foci after poly(I:C) transfection (200 ng/mL at 16h) in U2OS-A3B-Flag ± DOX. Red lines indicate the mean (Number of G3BP1 foci, n=250). P-values were calculated with a two-tailed Welch t-test. **D.** The level of G3BP1, G3BP2, and GAPDH were analyzed in the indicated proteins extracts by western blotting. **E.** Quantification of 250 A3B foci surface area from DOX treated U2OS-A3B-Flag (WT) transfected with siCTL or DOX treated U2OS-A3B-Flag G3BP1 KO transfected with siG3BP2 for 40h, subsequently transfected with poly(I:C) (200 ng/mL, 4h). Red lines indicate the mean. P-values were calculated with a two-tailed Welch t-test. **F.** A3B surface area quantification in U2OS-A3B-Flag (WT) + DOX knockdown for siPKR or U2OS-A3B-Flag G3BP1 KO + DOX knockdown for siG3BP2 and siPKR for 40h before transfection with poly(I:C) (200 ng/mL, 4h). Red lines indicate the mean (Number of G3BP1 foci, n=250). P-values were calculated with a two-tailed Welch t-test. **G.** Quantification of the number of HuR and FAM120A foci in U2OS-A3B-Flag (WT) + DOX or U2OS-A3B-Flag G3BP1 KO + DOX transfected with siG3BP2 for 40h before to be transfect with poly(I:C). Red lines indicate the mean (Number of cells, n=250). P-values were calculated with a two-tailed Welch t-test. **H.** Quantification of the number of HuR and FAM120A foci in U2OS-A3B-Flag (WT) + DOX transfected with siPKR or U2OS-A3B-Flag G3BP1 KO + DOX transfected with siG3BP2 and siPKR for 40h before to be transfect with poly(I:C) (200 ng/mL, 4h). Red lines indicate the mean (Number of cells, n=250). P-values were calculated with a two-tailed Welch t-test. Source data are provided as a Source Data file.

Supplementary Figure 9

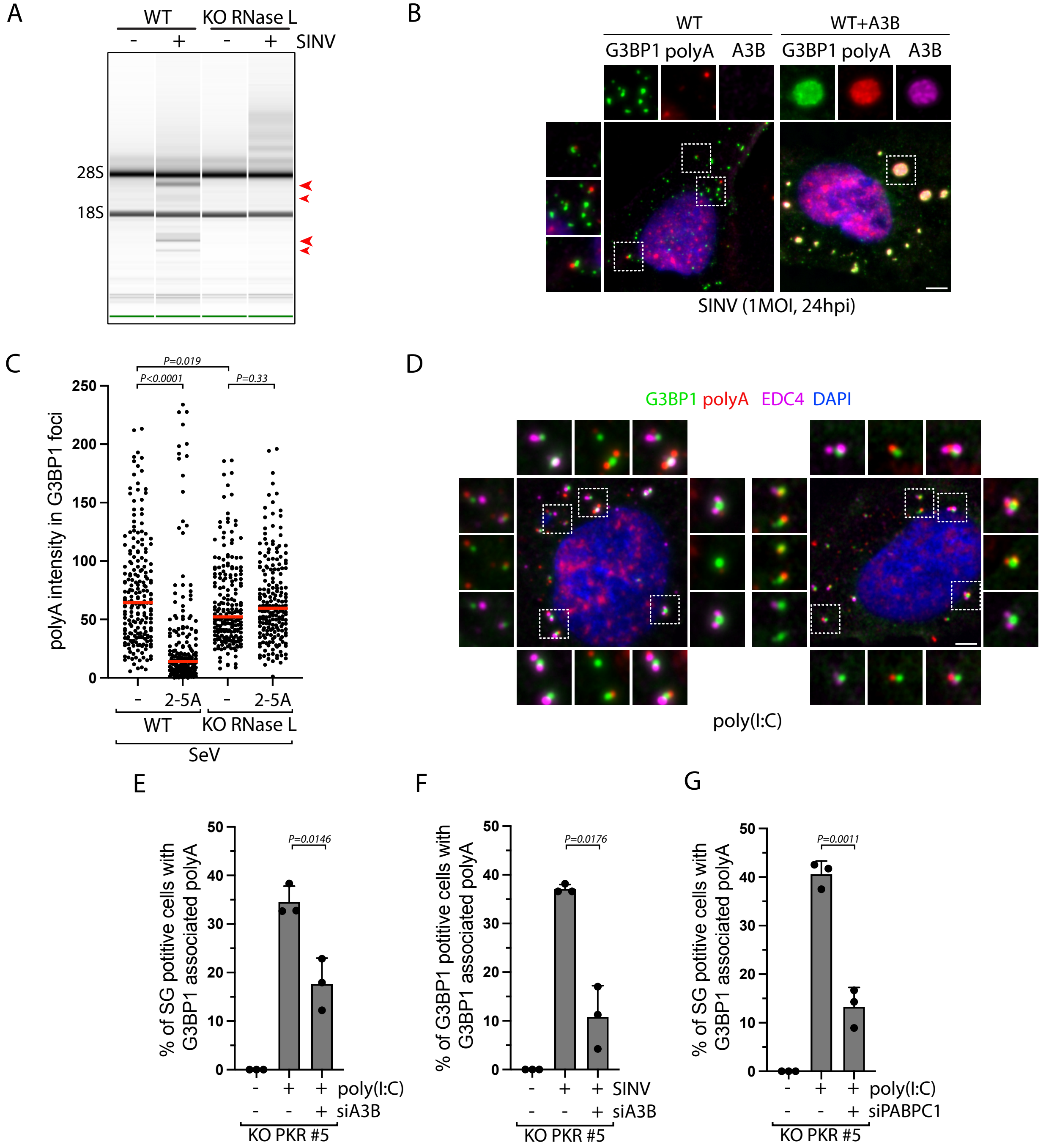

Supplementary Figure 9: A. Total RNAs from U2OS-A3B-Flag WT and RNase L KO cells infected with SINV (MOI=1, 24hpi) without DOX treatment were collected and analyzed for integrity by bioanalyzer. The red arrows indicated ribosomal RNA cleavage products. **B.** G3BP1, poly(A), and A3B (Flag) localization were monitored after SINV infection (MOI=1, 24hpi) in U2OS-A3B-Flag ± DOX by IF-FISH. Scale bar: 5 µm. **C.** U2OS WT and RNase L KO cells were transfected with 2-5A (20 µM) 8h after SeV infection. At 24hpi, poly(A) intensity (arbitrary units) was analyzed in G3BP1 foci. Red lines indicate the mean (Number of G3BP1 foci, n=200). P-values were calculated with a two-tailed Welch t-test. **D.** G3BP1, poly(A), and EDC4 were monitored by IF-FISH after poly(I:C) transfection (200 ng/mL, 4h). Scale bar: 5 µm. **E.** U2OS KO PKR cells knocked down with siRNA control (siCTL) or siA3B for 40h were transfected with poly(I:C) (200 ng/mL, 4h). The percentage of cells with G3BP1-associated poly(A) foci was quantified. Mean values ± SD (Number of biological replicates, n = 3). **F.** Quantification of percentage of cells with G3BP1-associated poly(A) foci in U2OS-A3B-Flag KO PKR cells without DOX treatment knocked down for A3B and infected with SINV (MOI=1, 24hpi). Mean values ± SD (Number of biological replicates, n = 3). **G.** Quantification of percentage of cells with G3BP1-associated poly(A) foci in U2OS-A3B-Flag KO PKR cells without DOX treatment knocked down for PABPC1 and transfected with poly(I:C) (200 ng/mL, 4h) transfection. Mean values ± SD (Number of biological replicates, n = 3). Source data are provided as a Source Data file.
