# Supplementary material for: APOBEC3B drives PKR-mediated translation shutdown and protects stress granules in response to viral infection
Source: Nat Commun. 2023 Feb 14;14:820. doi: 10.1038/s41467-023-36445-9 (PMC9925369; doi:10.1038/s41467-023-36445-9)
Supplement: Supplementary file 3 — Description of Additional Supplementary Files [file 41467_2023_36445_MOESM3_ESM.pdf]

Description of Additional Supplementary Files

File Name: Supplementary Data 1
Description: siRNA sequences

File Name: Supplementary Data 2
Description: gRNA sequences

File Name: Supplementary Data 3
Description: Antibodies

File Name: Supplementary Data 4
Description: qPCR primers
