# Supplementary material for: APOBEC3B drives PKR-mediated translation shutdown and protects stress granules in response to viral infection
Source: Nat Commun. 2023 Feb 14;14:820. doi: 10.1038/s41467-023-36445-9 (PMC9925369; doi:10.1038/s41467-023-36445-9)
Supplement: Supplementary file 5 — Reporting Summary [file 41467_2023_36445_MOESM5_ESM.pdf]

nature portfolio

Corresponding author(s): Rémi Buisson

Last updated by author(s): 2022/01/09

Reporting Summary

Nature Portfolio wishes to improve the reproducibility of the work that we publish. This form provides structure for consistency and transparency in reporting. For further information on Nature Portfolio policies, see our Editorial Policies and the Editorial Policy Checklist.

Statistics

For all statistical analyses, confirm that the following items are present in the figure legend, table legend, main text, or Methods section.

n/a	Confirmed	
☐	☒	The exact sample size (*n*) for each experimental group/condition, given as a discrete number and unit of measurement
☐	☒	A statement on whether measurements were taken from distinct samples or whether the same sample was measured repeatedly
☐	☒	The statistical test(s) used AND whether they are one- or two-sided<br>*Only common tests should be described solely by name; describe more complex techniques in the Methods section.*
☒	☐	A description of all covariates tested
☒	☐	A description of any assumptions or corrections, such as tests of normality and adjustment for multiple comparisons
☐	☒	A full description of the statistical parameters including central tendency (e.g. means) or other basic estimates (e.g. regression coefficient) AND variation (e.g. standard deviation) or associated estimates of uncertainty (e.g. confidence intervals)
☐	☒	For null hypothesis testing, the test statistic (e.g. *F*, *t*, *r*) with confidence intervals, effect sizes, degrees of freedom and *P* value noted<br>*Give P values as exact values whenever suitable.*
☒	☐	For Bayesian analysis, information on the choice of priors and Markov chain Monte Carlo settings
☒	☐	For hierarchical and complex designs, identification of the appropriate level for tests and full reporting of outcomes
☒	☐	Estimates of effect sizes (e.g. Cohen's *d*, Pearson's *r*), indicating how they were calculated

Our web collection on statistics for biologists contains articles on many of the points above.

Software and code

Policy information about availability of computer code

Data collection	qRT–PCR was performed in QuantStudio 3 (Applied Biosystems) using Applied Biosystems QuantStudio software (version 1.4.1).<br>Gel images were acquired using a Chemidoc MP (BioRad) and analysed with ImageLab 6.1 .<br>Immunofluorescence images were captured using a Leica DMi8 THUNDER microscope
Data analysis	Gel images were quantified with ImageLab 6.1 (Biorad).<br>Graphs were generated using Prism 9 (GraphPad).

For manuscripts utilizing custom algorithms or software that are central to the research but not yet described in published literature, software must be made available to editors and reviewers. We strongly encourage code deposition in a community repository (e.g. GitHub). See the Nature Portfolio guidelines for submitting code & software for further information.

Data

Policy information about availability of data

All manuscripts must include a data availability statement. This statement should provide the following information, where applicable:

- Accession codes, unique identifiers, or web links for publicly available datasets
- A description of any restrictions on data availability
- For clinical datasets or third party data, please ensure that the statement adheres to our policy

The source data underlying Figures 1 to 9, and Supplementary Figure 1 to 9 are provided as a Source Data file. All sequencing data were deposited in NCBI Sequence Read Archive, using the Bioproject Accession: PRJNA897945. A direct link can be found here: https://www.ncbi.nlm.nih.gov/bioproject/?term=PRJNA897945.

Human research participants

Policy information about studies involving human research participants and Sex and Gender in Research.

Reporting on sex and gender	not applicable
Population characteristics	not applicable
Recruitment	not applicable
Ethics oversight	not applicable

Note that full information on the approval of the study protocol must also be provided in the manuscript.

Field-specific reporting

Please select the one below that is the best fit for your research. If you are not sure, read the appropriate sections before making your selection.

☒ Life sciences ☐ Behavioural & social sciences ☐ Ecological, evolutionary & environmental sciences

For a reference copy of the document with all sections, see nature.com/documents/nr-reporting-summary-flat.pdf

Life sciences study design

All studies must disclose on these points even when the disclosure is negative.

Sample size	Sample sizes were chosen according to accepted standards in the field. Sample size was not pre-determined using statistics tolls. As indicated in the figure legends, minimal size of analyzed biological samples was "3". Statistical analysis (as described in respective figure legends) was used to calculate statistical significance of obtained results. The individual p-values are indicated in figures or in figure legends.
Data exclusions	No data were excluded.
Replication	All experiments have been repeated in multiple successfully independent experiments (3 times or more). All key experiments have been repeated in different cell lines.
Randomization	We had a limited number of biological samples. The analysis was self-normalized to the sample, so randomization of samples would not be a relevant method.
Blinding	As the analysis required comparisons against a known controls and knockdown targets were selected for their likely relevance to the biological pathway, blinding would not provide much reduction of potential bias in the analysis. However, performance and analyses of experiments were independently conducted by co-authors.

Reporting for specific materials, systems and methods

We require information from authors about some types of materials, experimental systems and methods used in many studies. Here, indicate whether each material, system or method listed is relevant to your study. If you are not sure if a list item applies to your research, read the appropriate section before selecting a response.

Materials & experimental systems

n/a	Involved in the study	
☐	☒	Antibodies
☐	☒	Eukaryotic cell lines
☒	☐	Palaeontology and archaeology
☒	☐	Animals and other organisms
☒	☐	Clinical data
☒	☐	Dual use research of concern

Methods

n/a	Involved in the study	
☒	☐	ChIP-seq
☒	☐	Flow cytometry
☒	☐	MRI-based neuroimaging

Antibodies

Antibodies used

Targets / Species and Antibody types / Clones / Antibody Dilution / Company and Catalogue number
APOBEC3B Rabbit monoclonal EPR18138 1/2000 WB Abcam: ab184990
APOBEC3F Rabbit polyclonal 1/1000 WB Homemade antibody raised against the last 30 residues of APOBEC3F (made by Dr. Harris laboratory)
ADAR1 Rabbit Monoclonal E6X9R XP 1/1000 WB Cell signaling #81284
DDX3X Mouse monoclonal 2253C5a 1/200 IF, 1/500 WB Santa cruz sc-81247
Edc-4 Mouse monoclonal H-12 1/200 IF Santa Cruz sc-376382
eIF2 Rabbit monoclonal D7D3 1/1000 WB Cell Signaling technology 5324T
eIF2a-pS51 Rabbit monoclonal E90 1/3000 WB, 1/500 IF Abcam (ab32157)
FAM120A Rabbit polyclonal 1/200 IF HPA 019734
Flag Rabbit polyclonal 1/500 IF. 1/3000 WB Sigma(#F7425)
Flag Mouse monoclonal M2 1/200 IF Sigma (A2220)
G3BP1 Mouse monoclonal 23/G3BP 1/500 IF, BD(#611127)
G3BP1 Rabbit monoclonal E9G1M XP 1/500 IF, Cell signaling (#61559)
G3BP2 Rabbit polyclonal 1/1000 WB proteintech (16276-1-AP)
GAPDH Rabbit polyclonal 1/20,000 WB EMD Millipore (#ABS16)
GFP Rabbit polyclonal 1/5000 WB Invitrogen #A11122
GFP Mouse monoclonal C163 1/1000 IF Invitrogen #33-2600
HA Rabbit polyclonal 1/1000 WB Invitrogen 71-5500
HuR Mouse monoclonal 3A2 1/500 IF Santa cruz- 5261
Matrin Rabbit polyclonal 1/4000 WB, 1/500 IF Bethyl: A300-591A-T
MOV10 Rabbit polyclonal 1/1000 WB, 1/300 IF Bethyl A3O1-571A-T
PABPC1 Mouse monoclonal 10 E 10 1/500 WB, 1/400 IF Santa Cruz sc-32318
PABPC1 Rabbit polyclonal 1/500 IF Abcam 21060
PKR Mouse monoclonal Clone 13 (RUO) 1/1000 WB BD Biosciences 610764
PKR-pT446 Rabbit monoclonal E120 1/500 WB Abcam ab32036
Puromycin Mouse monoclonal clone 12D10 1/500 IF EMD Millipore corp MABE343
SAF-A Rabbit polyclonal 1/1500 WB, 1/300 IF BETHYL: A300-690A-M
STAT1 (C-136) Mouse monoclonal C-136 1/1000 WB Santa Cruz-464
STAT1-pY701 Rabbit monoclonal 58D6 1/1000 WB Cell Signaling (#9167)
TIA-1 Mouse monoclonal G-3 1/200 IF Santa Cruz sc-166247
Vineculin Mouse monoclonal hVIN-1 1/5000 WB Sigma (V9264)
Rnase L (E-9) Mouse monoclonal E-9 1/1000 WB Santa Cruz sc-74405

Validation

Antibodies specificity against PKR, RNase L, G3BP1,and A3B were confirmed in this manuscript using knockout cell lines for the corresponding protein target. Antibodies specificity against MOV10, DDX3, MAVS, SAF-A, Matrin, ADAR1, A3F, eIF2a, eIF2a-pS51, G3BP2, PABPC1, HuR, STAT1, STAT1-pY701, PKR, and PKR-pT446 were confirmed using cell lines knockdown with specific siRNA for the corresponding target. Flag, GFP, HA antibodies were validated by transfecting a tagged protein (with GFP, Flag or HA) and compared to cell extract with transfection. Puromycin antibody was validate by comparing cell extract labeled or not with puromycin. TIA1, GAPDH, FAM120A, Vinculin, and Edc4 antibodies were validated by the manufacturer for western blots and referenced in other papers that are included in the manufacturer website.

Eukaryotic cell lines

Policy information about cell lines and Sex and Gender in Research

Cell line source(s)

U2OS, A549, Vero, and LLC-MK2 cell lines were purchased from either ATCC or Sigma-Aldrich. Knockout cell lines were derived from U2OS cell line as described in the method section.

Authentication

All cell lines were obtained from commercial repositories (ATTC, Sigma Aldrish). Upon receipt, the cell lines were expanded and frozen stocks were created. For the experiments described in this article, cell lines were not continuously kept in culture for more than 3 months. These cell lines were not authenticated

Mycoplasma contamination

All cell lines were tested repetitively for Mycoplasma contamination and all cell lines were tested negative for Mycoplasma

Commonly misidentified lines
(See ICLAC register)

No commonly misidentified cell lines were used in the study
